# The Nature and Treatment of Cancer

**Published:** 1846-04

**Authors:** 


### Art. VIII.

*The Nature and Treatment of Cancer.* By W. H. Walshe, m.d., Professor of Pathological Anatomy in University College, Physician to University College Hospital, and to the Hospital for Consumption and Diseases of the Chest.—*London,* 1845. 8vo, pp. 590.

In our last Number we gave a very brief, and promised a more complete, notice of this work. We now redeem the pledge, by laying before our readers a full analysis of its first two divisions, containing the general history and treatment of cancer: the concluding portion will occupy our attention on a future occasion. The fulfilment of this duty has been to us a most pleasing task. Unruffled in temper, and improved in mind, we have gone on from page to page, ever acquiring some new truth, or finding old ones more forcibly and clearly impressed on our understanding. And most firmly do we believe, that when our readers have perused this abstract, or, better still, have for themselves studied the original, they will return the same verdict that we have already delivered, and acknowledge it to be indeed "one of the completest and most valuable monographs of an individual disease that exists in medical literature."

The introductory chapter is occupied with the consideration of the place which cancer holds in the nosological scale, and the signification of the term itself. We shall notice these two points as briefly as may be.

1. Nosological position of cancer. Diseases may be arranged in two great groups—the *functional* and the *organic*. It is to the latter only that our attention is to be directed. In all these there is a demonstrable change either of form or of composition and texture of an organ, separately or conjointly. Congenital malformations and acquired changes of configuration occupy the first of these groups. The second consists of those in which there is a change in the physical or chemical attributes of the natural elements of the frame, and of those in which there is the addition of some new material to the original or fundamental tissues—in other words, an adventitious product.

Explained more fully, this term, "adventitious product," is held to mean, "any substance which, produced by or originating in connexion with the organism, neither forms part of its natural structure, nor results from its healthy manifestations of activity, while, at the same time, it differs more or less completely, in many or in few qualities, from all the natural constituents of the frame." The materials of which this substance is composed may be either physiologically inorganic or organic. To the former belong saline deposits, calculi, &c., and some proximate principles, such as oils, sugar, and albumen, which are exuded ready-formed from the vessels, and are either incapable by nature of assuming structure, or are rendered so by the special circumstance of their origin. The latter includes all those which are possessed of organization,—in a word, formations,—and are divisible into two sub-classes, blastemal and germ-formations, or parasites.

*Blastemal formations* depend for their existence on the immediate and direct access of nutritious matter from the blood of the parent organism, and are essentially characterized by cell-structure, their nature being determined by the attributes and end of the component cells. Thus, the cells may be incapable of maintaining their own existence, or of generating new cells previously to their destruction. They are consequently acted upon by the surrounding materials, dissolved in the fluids, or disintegrated and crumbled down. Cells of this kind may be called *evanescent*, and the formations they constitute *deposits*. Pus, tubercle, and colouring matters are examples of this kind.

Again, the primary cells may be able, before their destruction, themselves to generate, or indirectly to cause the generation of, new cells, which in their turn perform the same process. They may be called *non-permanent* and *indefinitely vegetating*, and their formations *growths*.

Lastly, the primary cells, though unable to generate others, may possess an inherent faculty of forming tissue, more or less closely resembling the natural structure and of continuing in this condition. They may be termed *persistent, non-vegetating cells*, and their formations *pseudo-tissues*.

We must speak now of the order of *growths*. The materials composing them are generally accumulated in some point or other of the body, where they gradually increase in quantity, pushing aside the structures among which they are developed. But there are some which besides this have a tendency to spread among the elementary molecules of those tissues, to cause atrophous destruction of existing particles, and prevent the evolution of similar new ones. This process is known by the name of *infiltration,* and the genus of growths which exemplifies it is *cancer.* In the following pages the truth of this position is attempted to be established.

With regard to the *signification of the term,* CANCER or CARCINOMA, we shall merely remark, that Dr. Walshe (while recognising three distinct species, encephaloid, scirrhus, and colloid, with several varieties) applies it generically to every formation of this class, whatever be its external appearance and in whatever condition of development or destruction it may be. In this, we think, he has done wisely. To multiply names is but to introduce confusion. The reader will find a very useful table at p. 10, exhibiting a synopsis of the species and varieties of cancer, with a list of synonyms; from this he may learn that names are even more abundant than real varieties of the disease. Having thus prepared the way for his inquiries, our author commences his task by the consideration of the *anatomy of cancer.*

ENCEPHALOID. This substance, so named from its resemblance to the brain, presents, on section, the appearance of an almost homogenous matter, of an opaque milky colour, variously dotted with pinkish spots. Its consistence is about that of the healthy adult brain, and, when torn through the surface, has a coarsely granular aspect. It consists of two materials, a stromal, or containing, and an intra-stromal, or contained. The stromal substance, generally denser than the other, divides the mass into loculi, lobules, and lobes. Its different divisions do not intersect, but describe curves, circumscribing spaces of a somewhat spherical form. It is occasionally of fibrous consistence, but most usually of a delicate cellular texture. But in almost all cases some parts of a tumour of this kind are harder and some softer than that above described, and this diversity is caused by the mode of arrangement of the cancerous structure; the hardest part being radiated, or finely granular, that of less consistence largely granular or lobulated, while the softest is collected into large lobes of almost diffluent pulp.

The pink tint is produced by the presence of numerous blood-vessels, varying in diameter from that of a hair to a line and upwards. The vessels are not confined to the stromal part; they penetrate the contained substance also. In some successful injections the whole mass has appeared to be formed of a vascular plexus.

Encephaloid is met with, either as a distinct tumour, or infiltrated among the elementary molecules of the tissue. Dr. Walshe doubts its being ever truly encysted, though it is occasionally surrounded by a pseudo-cyst of condensed cellular tissue. The tumours usually incline to a spheroidal shape, but this admits of many exceptions. The infiltrated, which is the less common form, is often seen in the liver, and is observed in all instances where the disease extends by continuity from one tissue to another. Encephaloid cancers acquire a larger size than almost any other morbid formation. On the other hand, the extreme of smallness is sometimes seen.

*Varieties.* There are several varieties of encephaloid, which are distinguished by the following names: *a. Mastoid,* the section of which presents the aspect of the boiled udder of the cow. *b. Solanoid,* which much resembles a sliced raw potato. It is of not uncommon occurrence in the liver. *c. Milt-like tumour:* this is rare. *d. Nephroid,* so-called from its resemblance on section to the kidney. In a post-peritoneal cancerous tumour, presenting all the characters of this form, as described by Recamier, microscopical examination showed the mass to be composed of granules, cells, and peculiar fibres, precisely as in Müller's *fasciculate* carcinoma, a proof that Dr. Walshe was correct in his views of the close alliance of these two varieties,—the nephroid and fasciculate. *e. Hematoid,* when the vessels are unusually abundant, the brain-like character being still retained. *f. Fungus hematodes.* This term should only be applied to tumours in which interstitial hemorrhage has produced sanguineous infiltration, or irregular accumulations of blood through its substance; and when, the integuments being ulcerated, a rapid development of fungating growths takes place from the exposed surface. As a name for the entire species it is most objectionable.

Scirrhus. This common form of cancer consists also of a stromal and an intra-stromal substance; but in many cases it requires close inspection, in different lights, to distinguish between them. The white intersecting bands, so often spoken of, are an accidental condition produced by thickening and cancerous infiltration of some element of the affected organ, such as lymphatics, or, in the mamma, lactiferous tubes. The stroma has a tendency to an irregularly rectilinear arrangement, whence the loculi incline to the rectangular shape. They are of small size. Dr. Walshe is confident that those writers who have described the loculi as large and spheroidal have mistaken fibrous tumours for true scirrhi.

The proportion between the stromal and intra-stromal matter varies according to the period of development of the tumour. In a very early stage the former predominates, and the mass resembles fibro-cartilage—so closely indeed that M. Cruveilhier believes the appearance of the cancerous juice on pressure is the only diagnostic mark. According to our author, the two may be distinguished by the arrangement of the stroma—rectilinear in scirrhus,—curvilinear in fibrous tumours.

The section of a scirrhus has a peculiar semi-transparent glossiness; it is of a bluish-white colour when the mass is most firm—of a lilac-yellow, pale dirty fawn, or grayish, when there is much intra-stromal matter.

One of the chief characteristics of this form is its extreme hardness. This is most decided while the tumour is still *in sitû;* after removal it often becomes soft and somewhat flaccid from the absence of blood and the *turgor vitalis.* The fluid which may be expressed from very solid masses is thin and watery; that from the less firm is thicker and more opaque.

Scirrhus is not strikingly vascular. It has even been denied that it possesses any vessels; but this is a mistake. The supply, however, is irregular and unequal. It is deposited either as a tumour or infiltrated.

The latter form is not uncommon in the brain, and is its most usual state of existence in the uterus and the bones. Dr. Walshe has never seen a scirrhus tumour invested by a true cyst. The morbid growth rarely acquires a large size.

*Anatomical varieties.* 1. *The pancreatic sarcoma* of Abernethy is regarded by Dr. Carswell and some other pathologists as a variety of scirrhus. Our author has great doubts regarding the true nature of the tumour referred to, and believes the term would be more correctly applied to some form of encephaloid, or to the pultaceous alveolar carcinoma of Cruveilhier. 2. Dense scirrhi, of an unusually shining aspect, and bluish-white colour on section, are distinguished by Recamier by the name *chondroid.* 3. The term *lardaceous tissue,* much employed by French pathologists, is applicable to the infiltrated form of scirrhus, where a section of the diseased part resembles the boiled rind of bacon. 4. In those rarer scirrhi, which present on section an evident appearance of bands, the surface occasionally resembles a cut turnip, and such are denominated *napiform.* 5. The adjective *apinoid* is applied to those which, when divided, resemble the section of an unripe pear; the similitude being produced by the dissemination of comparatively opaque, almost buff-coloured, spots through a more translucent ground of a very pale yellowish-lilac tint. As development proceeds, the opaque matter gradually increases and alters the appearance of the surface. It is the most ordinary condition of mammary scirrhi. Müller has described it as a distinct species, under the name of *carcinoma reticulare.* 6. Lastly, in some rare cases scirrhi are found in the *hematoid* condition, and these present the same peculiarities as that variety of encephaloid.

Colloid. This form of cancer presents on section the following appearance:—The surface is divided into a vast number of distinct loculi, of an oval or rounded shape, varying in size from that of a grain of sand to the largest pea. The septa are fibrous and of a pretty uniform thickness. The loculi sometimes form shut sacs, sometimes communicate with each other. They are filled with a semi-transparent, tenacious, and clammy matter, which has the physical properties of soft jelly, and gives a greenish yellow colour to the section. The consistence of the tumour is usually about that of firm cheese, but it may be much harder. It is a remarkable character of this structure that wherever and in whatever form developed, the appearance of properties of the gelatiniform substance are exceedingly uniform in every part of its extent, so long as destruction has not set in. Little is known regarding its vascular condition. Colloid is found as a distinct tumour, and infiltrated. The latter is most common in the stomach, the organ retaining its natural shape. It is also met with in the intestines and omentum. Disseminated nodules never occur in the parenchymatous organs. Colloid growths acquire considerable bulk. In the infiltrated form it sometimes extends over more than two thirds of the anterior and posterior surfaces of the stomach.

It can scarcely be said that there are any true varieties of colloid. The contained matter is somtimes pearl-like, from the presence of a peculiar kind of cholesteric fat, called by Müller *cholesteatoma.* M. Cruveilhier describes specimens in which the matter was opaque, of yellowish hue, and tallow-like aspect, with granular fracture and feel, and the chemical constitution of casein: this constitutes his *areolar pultaceous cancer;* but Dr. Walshe considers that it is more closely allied to encephaloid. Colloid is sometimes developed in a basis of scirrhus. In cases of this kind the appearance presented has been mistaken for softening of the scirrhus mass.

CHEMISTRY OF CANCER. But little yet is known upon this point, though the inquiries of different observers, and especially of Müller, have assigned cancer a place among the protein formations. It belongs essentially to the albuminous variety, though both fibrin and casein enter slightly into its composition. There is some difference of opinion as to the presence of gelatine; but the weight of evidence leans to the negative. Its occasional discovery has been very plausibly attributed to the intermixture of ordinary cellular tissue. Scirrhus is distinguished from encephaloid by containing a less quantity of albumen, by being wholly deficient in "osmazome," and by having nearly twice as large an amount of inorganic salts, the proportion being 37·25 per cent. in scirrhus, 19·10 per cent. in encephaloid. Müller discovered casein in his carcinoma reticulare, and considers it a very uniform constituent of mammary carcinoma. He conceives that its presence does not depend upon the contents of the lactiferous tubes, because it also exists in the morbid product in other organs. Spirits of wine, which renders scirrhus and encephaloid opaque, has no effect on the transparency of the jelly-like matter of colloid. Müller could not find any trace of gelatine in it; nor does it contain casein, excepting in the so-called pultaceous variety. There is some osmazome; but the chief constituent is albumen. There is also present a small quantity of a matter analogous in many respects to the salivary principle (ptyalin).

The ultimate microscopical cells of cancer are insoluble in cold and boiling water, and are not seriously affected by acetic acid—characters distinguishing them from the red and white corpuscles of the blood, and from pus corpuscles. Crawford found the *ichor and discharge* alkaline, and ascertained the presence of free ammonia; he also detected sulphuretted hydrogen. Ploucquet states that the matter sometimes effervesces with alkalies, sometimes with acids. The question seems yet undecided.

PHYSIOLOGY OF CANCER. This is, perhaps, the most interesting portion of our inquiries. The Physiology of Cancer includes the consideration of its Origin, Growth, Decay, Elimination, Cicatrization, and Local Regeneration.

I. *Origin.* Our space will not permit us to follow Dr. Walshe in his review of the different theories—some most absurd, others more specious and truth-approaching, which have, from time to time, been promulgated regarding this mysterious subject; though it is most ably executed, and will well repay an attentive perusal. We must confine ourselves to an exposition of the author's own peculiar creed.

There are three points really distinct in nature, which have invariably been more or less confounded by investigators of this complex question. These are—A. The nature and properties of the formative material in which the new mass originates; B. The seat or locality in which it originates and germinates; C. The process by which this formative material is itself generated.

A. *Nature of the formative material.* Cancer, as we have seen, originates in a fluid denominated blastema. Its characters when first produced are unknown ; as existing in the substance of growths already evolved, and forming the matrix for additions, it in nowise differs from the blastema of pus, or of the natural tissues—being fluid, slightly viscid, semi-transparent, homogeneous, and totally devoid of solid particles. Its chemistry has not been ascertained. In all probability it is essentially composed of liquor sanguinis modified in its vital qualities. What the precise nature of this modification is we know not. How and where the change is effected is also doubtful; probably the peculiar attributes are mainly impressed upon it, while still mixing with the circulating fluid, though in some degree they are acquired during the process of filtration through the vascular parietes. Whether extravasated blood, unaltered in its apparent physical characters, is capable of acting as a blastema for cancer, is yet undetermined.

B. *Seat of germination.* There are three possible situations in which cancer-blastema, being derived from the blood, may be evolved, viz., in the interior of the vessels, in the substance of the vascular walls, or outside the vessels. In very few cases is there any evidence in favour of the first two ; the last is by far the most common locality ; and in the vast majority of instances, the site of the deposit is the inter-vascular interstices of the various tissues and organs. Where extra-vascular tissues are destroyed by the advance of adjacent cancerous disease, Dr. Walshe believes the process is effected by the imbibition of the blastema thrown out by the vessels of juxta-posed vascular tissue ; the fluid which has thus penetrated, subsequently passing through the same series of changes as if infiltrating a vascularized tissue.

C. *Nature of the process by which the formative material is generated.* The account given above sets at rest the *vexata questio* of the nutritive or secretive origin of cancer; for the exudation process, by which cancerous blastema, as all other unhealthy materials, is thrown out of the vessels, is neither one of secretion nor of nutrition, but may be a substitute for both.

II. *Growth.* The ultimate solid constituents of carcinoma are granules ; nucleated cells of spherical, elliptical, irregular, or caudate shape ; free nuclei, and fibrillar substance: with these is associated a variable quantity of the semi-transparent blastema. Capillary vessels are always discoverable ; and fat globules too are constantly present. Compound granule corpuscles, melanic matter in the form of cells and free granules, saline matter, and certain other substances, occur as accidental and contingent elements. For a full description of these, we must refer to the original; only observing, with regard to the vessels, that they are of two classes, some being found by the looping or extension process, from the vessels of the surrounding tissues, and others originating *de novo* in the morbid substance; these contained blood, exhibiting an oscillatory movement, independent of the general circulation. This last fact is of importance as showing, what has been hitherto entirely overlooked, that out of the same fluid blastema, and at one and the same time, are produced cancer-cells, and cells destined to form structure closely similar in all its properties, to one of the natural tissues of the body, viz. blood-vessels.

How, then, are the cells formed? Two methods of development are described. In one, the cell is a molecular vesicular body from the first, and produces within it a secondary body, the nucleus, which is the embryo of a future cell: in the other, granular matter is first precipitated from the blastema, and this constitutes a nucleus, around which the cell forms. Dr. Walshe believes that both may be seen in the cancer. Caudate cells are produced by elongation of opposite points of the circumference of the spherical cells. They eventually pass into the state of filaments, and become the elements of fibrous structure. Our author believes that the diagnostic importance of these cells has been greatly exaggerated, and that some at least originate from non-cancerous spherical cells.

The tumour being thus formed at first, its *enlargement* is effected in the following manner:—It is supplied with nutritive materials by vessels precisely in the same way as the natural tissues, and from the matter thus afforded, there is a continuous cell-generation, the mode of which differs in different cases. Thus, in some, the primary cells contain within them the nuclei of a second generation of similar bodies, which, in their turn, possess the like procreative faculty: and the process may be carried on *ad infinitum,* so long as there is a supply of blastema. Of this, the *endogenous mode,* we have an example in colloid. In other cases, as in Scirrhus, and most commonly in Encephaloid, the germs of secondary cells are not formed within the primary ones, each succeeding generation being formed external to preceding ones. This is the *non-endogenous mode.* It is probable that the locular arrangement, and the general shape of the whole mass, are caused, in some measure at least, by this manner of increasing.

III. *Decay.* A tendency to destruction is inherent in all formations of this genus, but the way in which it is produced has been differently explained. According to some, it results from gangrene, produced by obstruction of the venous trunks; while others regard inflammation as the exciting cause. Dr. Walshe believes that both views are occasionally correct, but that in some instances, the softening process can only be explained by considering the change, similar in nature to that which has been shown by Mr. Gulliver, to occur in fibrine stagnating in the vessels. Colloid is in a different position; its destruction is produced, either by a gradual wearing out of the stromal matter, and subsequent escape of the contained jelly, or by the rupture of the containing parts from extremely active growth of the sub-cells.

We must pass by the three remaining sections of this chapter; nor can we do more than direct attention to the succeeding one, which describes the local changes to which cancerous tumours are liable, and the effects they produce on surrounding parts. But the next chapter, on the pathology of cancer, is one of such importance, as to demand a more full investigation.

GENERAL PATHOLOGY OF CANCER. It has been long known, that some parts of the body, as the uterus, female mamma, stomach, liver, &c. are more prone than others to cancerous disease, but we have as yet scarcely any numerical estimates. The reader will find two documents of this kind in the work before us (pp. 92-3). The causes of this predisposition have been the subject of much speculation; but the question remains totally undecided. It is quite evident that age and sex exert a great influence on the localization of the disease; and it is a curious fact, that particular parts of organs are more liable to be attacked than others, as, the upper third of the œsophagus, the pyloric end of the stomach, the middle lobes of the brain, &c.; and that, when one only of double organs is affected, it is most usually the right.

*Primary cancer* has been found in all organs and tissues of the body, excepting tendon, ligament, the intervertebral tissue, probably articular cartilage, and the spleen. Still, more extended observation may prove, that even these can afford a primary nidus to the disease. Dr. Walshe has not met with any cases of *secondary* development (excluding those in which it occurs, from extension of the disease by continuity of tissue) in the eye, ear, or penis; and it is rare in the testis, mamma, uterus, tongue, lips, ovaries, prostate gland, and the entire tract of the alimentary canal. The parenchymatous viscera, the lymphatic and venous systems, the bones and the skin are its chosen seats; and it should be particularly noticed that it is in the peripheric portions that the secondary deposit takes place, the disease, when primary, choosing a deeper-seated locality; and that, in respect of double organs, both are almost invariably implicated at one and the same time, which rarely, if ever, occurs under other circumstances.

The species of the disease also influences its localization. *Encephaloid* has the widest range, and the seat of scirrhus is nearly as extensive, while it is doubtful if colloid has been really found in other situations than the stomach and adjacent glands, the small and large intestine, the omentum, female mamma, and spinal marrow. The different species affect, likewise, a special preference for certain localities. Encephaloid is most common in the testis, lungs, kidneys, spleen, and meninges; scirrhus in the uterus, female breast, stomach, lower lip, and skin; scirrho-encephaloid is *per eminentiam* the hepatic cancer; while colloid infests the alimentary canal and omentum. The reason of all this is utterly unknown. In many cases cancer exists in several parts of the body at the same time; but the observations of Dr. Walshe have led him to believe that this occurrence is less invariable than is commonly imagined. Where it does take place, the dissemination may have been produced by a successive, or by a simultaneous process of development. We need only notice the first of these, for the second requires no explanation, and is probably more common than is generally thought.

*Successive dissemination.* Here the disease originates in a single organ or tissue, whence it spreads to a multitude of parts, which are then said to be affected with *secondary* or *consecutive* cancer. When the parts thus attacked are circumjacent, the process is affected by what is called infiltration, and this may take place through tissues naturally continuous, or artificially rendered so by adhesions, but it has been also observed where the parts were merely in juxtaposition or close proximity. The infection of distant organs must be explained in another way. When direct lymphatic communication exists, it is through these vessels that the contamination is transmitted; when there is no such communication, it is more than probable that the veins are the conducting channels, as in cases of secondary purulent deposits. This doctrine has been opposed on various grounds, but we believe it is essentially true, and conceive that our author, to whose pages we again beg to direct the reader's special attention, has most successfully refuted every objection. Of the three species of cancer, encephaloid is perhaps the one which has the greatest tendency to affect other organs, though its pre-eminence in this respect is disputed by scirrhus. Colloid certainly holds the lowest rank. Secondary carcinoma is, in the majority of cases, of the encephaloid species.

*State of the solids and fluids generally.* There is no particular condition of the *solids* sufficiently constant to deserve the name of a "secondary lesion." But in protracted cases of the disease the mass of the solids diminishes. Louis has ascertained that the mean size of the heart is less than in persons who have died of any other disorder, and that the aorta at its commencement has also a smaller diameter. More is known regarding the fluids. The quantity of blood gradually decreases, and it acquires a peculiar clamminess or stickiness. Andral has detected pus-corpuscles, and in one case elliptical, finely-granular lamellæ, much larger than pus-corpuscles, and with a far more regular outline than mere patches of albumen. As the disease advances the proportion of fibrin increases, while that of the red corpuscles diminishes. These changes produce a tendency to passive serous effusions. Andral has detected pus-corpuscles in the chyle of the thoracic duct. The urine does not undergo any specific alterations.

*Symptomatology.* It remains yet to be ascertained whether there exist any *prodromata,* and if so, what is their nature. When cancer is once formed, the *local symptoms* are tumour of variable size and form (this exists in all cases, even when the morbid matter is infiltrated) : alteration of the shape of organs and parts ; pain, but this is exceedingly uncertain, being often trifling, and sometimes altogether absent; hemorrhage, in some cases the first appreciable sign of the disease; and derangements of functions, which of course vary according to the organ affected. In a few exceptional cases the malady runs its course without the appearance of any *general symptoms;* but most usually the constitution suffers in a marked and peculiar manner. There is fever of low type; gradually failing appetite; distressing thirst; often nausea and vomiting; irregular action of the bowels; progressive emaciation; a most peculiar straw-coloured and waxy appearance of the skin and mucous membranes, with semi-transparent puffiness; extreme debility; and insomnia. The bones are often affected, and become remarkably fragile. The intellectual faculties lose their clearness towards the close of the disease; the ideas are sad and gloomy; and the temper more or less morose. Such are the more prominent characteristics of the *cancerous cachexia;* but they do not appear with perfect uniformity. Their causes must probably be sought for in some morbid condition of the blood, though different from that which produces the cancerous diathesis. Were it the same, both phenomena would invariably coexist, which is not the case.

Course. Cancer is essentially a chronic disease, but occasionally it proves fatal in a very short period. Dr. Walshe relates at p. 131 a very interesting case in which death occurred in two months from the outset. The progress is usually gradual and uniform, but it is sometimes intermittent, giving rise to false hopes. In some instances the disease remains latent throughout its entire course.

*Terminations.* Death is the most common end ; but now and then a more favorable result is brought about, the disease being arrested for an indefinite period, or cured. Of cure as the effect of treatment we shall speak elsewhere ; in this place it is merely necessary to observe that it sometimes takes place spontaneously—the tumour undergoing resolution, or being destroyed by suppuration, or mortification. In a few very rare cases there has been a perfect and permanent cicatrization of the ulcerated parts.

*Duration.* We have few data as yet to determine this point. Our author has collected 56 cases, MM. Herrick and Popp 50, and M. Leroy d'Etiolles 1172. These three series give a mean duration of 39·43 months. Cases interfered with by surgical operation are not included in the above. Of course the commencement of the disease is dated from the first appearance of symptoms, and the results are, therefore, merely approximative. Of the three species encephaloid runs the shortest course. Sex does not appear to have much influence. Its progress is slower in old than in young persons.

*Frequency and mortality.* From the Registration Reports during the last 5 years, it appears that a mean number of 2644·66 persons die annually of cancer in England and Wales, or as closely as possible 176·83 of every million living; and that 8045·83 per million of all deaths are due to this disease. Mr. Wylde gives 1 in 305·23 of the deaths from all causes as the proportion in Ireland during ten years. The tables furnished by our author seem also to show an increase in the number of deaths during the last five years, but he is inclined to attribute this appearance to the greater accuracy of the registers, and the more perfect diagnosis of the disease. It does not appear that *season* exerts any influence on the mortality.

*Etiology. Specific causes.* There is no proof that the disease has ever originated from infection or contagion. It cannot be produced by inoculation, and the repetition of Langenbeck's experiment of injecting cancerous matter into the veins has given only negative results. *Predisposing causes.* It is the generally received, and probably correct, opinion that the disease is transmitted from parents to their offspring, but we have no positive proof of the fact. Our author gives some valuable tables illustrative of the influence of age, from which it appears that the mortality in both sexes steadily increases with each succeeding decade until the eightieth year; thus showing the inaccuracy of the common opinion that the tendency to cancer reaches its maximum between the 35th and 50th years. This idea has probably arisen from the sudden and enormous increase in the deaths of females in the last-named period (see a most instructive diagram, p. 50)—a result perhaps correctly attributed to the declining activity and cessation of the genital functions. As corroborating this view, Dr. Walshe quotes a curious document, showing that in Sweden and Finland the intensity of fecundity, which had been pretty uniform from æt. 25 to 40, suddenly falls to $\frac{1}{5}$th of its previous amount from æt. 40 to 50. Encephaloid is the most common species in early life ; but its pre-eminence in this respect has been much exaggerated. Of the influence of *sex* there can be no doubt. The mortality among females is about $2\frac{3}{4}$ths greater than among males. Our author believes that persons of the *sanguineous temperament* are most prone to the disease. It is impossible to decide with accuracy upon the effect of marriage or celibacy on the generation of the disease. Previous maladies, depraved habits, and too sustained intellectual labour, have all been assigned as causes, but without sufficient proof. There are, however, some grounds for believing that mental affliction has in many cases had some effect in inducing the malady. The influence of different occupations and stations in life is also yet to be determined; but Dr. Walshe has clearly shown that it is a mistake to imagine that a country life confers any immunity (p. 163). Cancer is most common in Europe; it is much rarer in Asia and tropical America, and is specially infrequent in Africa. Has, then, civilization any effect in producing the disease? Data are wanting for a satisfactory answer; but the bias of evidence is towards the affirmative. Mechanical injury is often one *exciting cause* of the disease; but the relative frequency of its operation is unknown. In some cases a state of irritation precedes the appearance of the disease; but it must ever be remembered that without a constitutional predisposition all these agents are inoperative; for, as our author elsewhere most truly remarks, "a cancerous tumour under all circumstances, even should it remain single and stationary for years, is but the local evidence of a general vitiation of the system."

Affinities and Nature of Cancer. In our last number we quoted in full Dr. Walshe's most philosophical views regarding the latter of these points; and in reference to the former we have only space to observe that the reader will find in the original a clear exposition of the characters which separate cancerous productions from all other growths; with some of which they have been often confounded. And this brings us to what is, after all, the most important branch of the whole subject—the treatment.

Treatment of Cancer. Little can be said regarding *prophylactic* treatment, for the simple reason that we know scarcely anything of the prodromata of the disease. But it is quite evident that individuals who belong to a family, any members of which have become the subjects of cancer, should adopt all those medical and hygienic rules which experience has shown to be most efficacious in invigorating a debilitated constitution.

*Curative treatment.* Before referring in detail to the various expedients which have been adopted for the cure of cancer, we may premise once for all that almost every remedy, every plan has been extravagantly lauded, or unduly depreciated. Of these extreme opinions we shall take no notice; our object being to lay before the reader a succinct account of the conclusions to which our author has arrived, after most careful investigation and much experience; and which, moreover, we think exhibit the evidences of sound judgment and impartial discrimination.

*Drugs.* Under the long-sustained use of *conium* the progress of the disease has, in some cases, been permanently arrested; but it is difficult to persuade patients to persevere in its employment. In Dr. Walshe's hands it has failed to do more than alleviate pain and irritability. *Belladonna, aconite, henbane, prussic acid,* and other narcotics are less worthy of trial, excepting as palliatives. *Sedum acre* merely quiets irritability, and often disagrees. Benefit may sometimes be derived from the local and internal use of *antacids*, especially *ammonia*. In many cases, local relief and decided general improvement of health have resulted from the employment of various preparations of *iron*. The *iodide* is perhaps the salt which offers the greatest promise of advantage. *Gold* has been much used in France, and occasionally with good effect. *Copper* and the *chloride of barium* seem unworthy of trial. *Mercury*, given so as to affect the gums, is a decidedly hazardous remedy; in minute alterative doses it is more safe, and has been well spoken of. *Iodine* is a much more eligible remedy, of the alterative class, and when combined with arsenic, in the form of the iodide of that metal, is perhaps the remedy which, in conjunction with external treatment, especially compression, offers the most chance of success. When given in doses of $\frac{1}{15}$ to $\frac{1}{12}$ grains twice daily, two hours after eating, it is well borne, and may be continued without risk for several months. Its effects are—unusual perspirations, with dryness of the fauces and alimentary canal; sometimes, but rarely, slight headache; decrease of pain; arrest of growth in the tumour, if not actual diminution of bulk; and general improvement of the health. *Animal charcoal, the flesh of the gray lizard,* and *cod and skate liver oils,* have been recommended. The latter are worthy of trial.

*External remedies.* Leeches are only useful in the very earliest stages (chiefly to help in the diagnosis), and to remove accidental inflammation in the adjacent textures. A continued reiteration of the process is a most mischievous and senseless proceeding. When a tumour is adherent to the skin, the practice is quite inadmissible. Ligature of the nutrient arteries has been occasionally advantageous. Blistering is a hazardous expedient; and the evidence in favour of electricity and galvanism is quite defective. Dr. Walshe has no experience of the effects of mercurial inunction and plasters; but he speaks favorably of the iodide of lead ointment, and prefers it to all other preparations of iodine. M. Tanchou strongly recommends the application of many of the substances above noticed in the dry form in *sachets*. When too strong in themselves, he mixes them with some inert powder. The only advantage of this plan is the change in the form of our applications. Of *compression*, the best external method of treatment, and one, moreover, which does not interfere with any other outward application, we have already spoken in our last Number, and to it we need not recur.

*Subcutaneous incisions* have been performed by M. Tanchou, with a view of preventing extension of the disease and promoting absorption. The only possible effect of such a proceeding is to make matters infinitely worse than they were before. Various applications have been used for the purpose of altering the character of exposed cancerous surfaces. M. Senebier's experiments show that *gastric juice* is capable of producing considerable temporary amendment. In one case under its employment an ulcer, which originally measured three inches in diameter, was reduced to the size of a sixpence. He recommends the gastric juice of the ox, as being always readily procured. *Carbonic acid* is another agent of this kind, but of inferior efficacy. *Lead* has been highly vaunted; but its powers appear to be merely sedative and anodyne. As exercising this action in a marked manner, Bayle recommends a combination of six drachms of litharge of gold, six drachms of vinegar, and two ounces of olive oil, to be spread in a thin layer on the ulcerated surface. *Tincture of iodine* improves the aspect of the sores and the character of the discharge, but can do no more. *Petroleum, tar-ointment,* and *creosote* are of less value. *Turpentine* was at one time much employed, but has deservedly fallen into disuse; and the *terchloride of carbon* has been found to have no active influence of any kind.*

The employment of *escharotics* in the cure of cancer is of very ancient date. The actual cautery was the first used, but is now rarely had recourse to; and of all the potential caustics, *arsenious acid* and the *chloride of zinc* are those which most deserve attention. Not a few cases are on record in which the disease has been effectually removed by the former; but its application is not unattended with danger, especially when it is requisite to repeat the process. For this reason the latter should, we think, be made choice of; and the cases in which it can be advantageously employed are, superficial cancerous ulcers of the skin, carcinoma appearing on the cicatrix after operation, and when some suspicious substance has been accidentally left behind.

*Palliative treatment.* The objects sought by palliative treatment are, modification of the course of the disease locally and generally, and relief of symptoms. Cold applications and evaporating lotions are indicated when the affected part is hot, full, and tense. Hemlock poultices, bruised carrots, the aqueous solution of opium, fomentations of stramonium leaves, and other sedatives may be used to allay pain. Vegetable charcoal and the chlorides of lime and soda will remove fætor. Decoction of cinchona with tincture of myrrh is serviceable when the surface is flabby and disposed to bleed. The temperature of these dressings may in a great measure be regulated by the feelings of the patient; but it should be remembered that *warm* applications are really detrimental. If severe hemorrhage set in, and do not yield to the internal use of acetate of lead, gallic acid, or ergot of rye, combined with the application of cold or the oil of turpentine externally, more active measures must be resorted to. When depending on capillary effusion, *pressure* is the most likely means of stopping the flow of blood; when from the perforation of an artery the vessel should be tied outside the morbid growth. And while these local measures are enforced, the patient's health and strength should be kept up to as high a standard as possible, by the use of general means. Pain must be soothed and sleep procured, for nothing wears the sufferer out so much as want of rest; and for this purpose opium or morphia is the best means; but the other narcotics may be employed as a change. The *diet* should be simple and nourishing, but unstimulating, except under particular circumstances. The starving system has been recommended and rigidly pursued; it is, however, a cruel and most useless torture—the body will waste, while the growth is luxuriantly flourishing. The *hygienic condition* of the patient must be carefully attended to. Regular moderate exercise, cheerfulness of mind, avoidance of intellectual labour, and the cares of business, should as far as possible be secured. In some cases,

* We have employed this substance both internally and externally in many cases, with the view of alleviating neuralgic pains, but have invariably failed in producing any effect. (REV.)

change of air and scene may be beneficial; and in the early stages of some cancers, especially of the breast and uterus, a course of mineral waters, taken at their source, as the Kraenchen-brunnen at Ems, and afterwards the Paulinen at Schwalbach, would be advisable.

*Treatment by ablation.* The removal of the morbid mass may be accomplished by cutting instruments, ligature, potential caustics, or congelation, or by inducing sphacelus. The first method is the one that has been most generally adopted, and concerning which it is of the first importance that we arrive at some definite conclusion. Our author accordingly devotes considerable space to its investigation, and we can state that nowhere will the student or practitioner find so able an exposition, or so critical and judicious an examination of this much agitated question as in the pages now before us. To accompany him throughout his whole chain of reasoning would occupy more space than we can spare; but we beg the reader's special attention to the following series of propositions, which, as it appears to us, he has logically deduced from his premises. They refer, be it observed, to the operation as a *curative measure.* We give these propositions in the author's own words; but it is due to him to state, that we do not here give *all* his words. To almost all the propositions are appended explanations, illustrations, and qualifications, which give additional force to the conviction their simple enunciation must produce.

"1. The removal of a cancerous tumour *may* cure the disease.

"2. No combination of circumstances ensures the patient, who submits to the excision of a cancerous growth, from return of the disease.

"3. Scarcely any combination of circumstances renders recovery from the operation, and prolongation of life, a matter of absolute impossibility.

"4. The existence of cancerous contamination of the lymphatic glands renders failure of the operation as nearly as possible a matter of absolute certainty.

"5. Where any portion of the morbid growth is accidentally left behind, the disease runs a materially more rapid course than if it had not been interfered with surgically.

"6. Should the cancerous diathesis exist, (no matter whether its manifestations have become actually apparent or not,) removal of a tumour inevitably determines its outbreak.

"7. When cancers in a state of active growth are cut out, the invariable result is reproduction and increased energy of progress of the morbid mass.

"8. Cancers, the exciting cause of which has been some local injury, are probably less prone to return after operation than those which have sprung up spontaneously.

"9. The success of excision in individuals belonging to a family of cancerous taint is even below the average.

"10. Reproduction of the disease becomes almost inevitable in cases where the skin or textures intervening between the tumour and integuments participate in the cancerous affection, or where these structures are, though not cancerously, still morbidly affected in consequence of the juxtaposition of the new growth.

"11. Even simple adhesion of the new formation to the part in which it grows, or to the subjacent textures, renders failure almost a matter of absolute certainty.

"12. The existence of the cachexia renders perfect and permanent recovery infinitely improbable, if (prop. 3) not impossible.

"13. With each repetition of the operation in the seat of original formation, the chances of return of the disease increase to an incalculable degree.

"14. The removal of two cancers simultaneously, or immediately after each other, may be considered a procedure which will almost of necessity be followed by return of the disease.

"15. The probabilities of effecting a cure are said to vary with the species of cancer.

"16. The removal of cancerous growths is more prone to be followed by relapse when the disease occupies certain organs or tissues than others.

"17. In cases where cancerous substance is simply superadded to preexisting encysted structure, the chances that relapse will not occur are above the average, provided the entire of both morbid products be removed.

"18. Cancers which have spontaneously, or through the influence of treatment, ceased to enlarge, to cause local suffering, or general disturbance—in fact, become quiescent—have sometimes been cut out, and the operation been followed by rapid reproduction of the disease, with aggravation of the local and general symptoms and early death.

"19. Organs, as for instance the breast, have sometimes been removed for affections presumedly cancerous, but in reality of the most innocuous character (e. g. cysts, and even abscesses): in such instances the operation has occasionally proved fatal." (pp. 231-6.)

We give also, in the author's own words, the general inferences which seem to us, as well as to him, to flow of necessity from these premises:

"First: in as much as the number of permanent recoveries is infinitely small, and as no combination of circumstances, however favorable, protects the patient from relapse,—*the operation cannot in any individual case be recommended as likely to cure the disease.* Secondly: in as much as no operation by excision is performed without the chance of some of the diseased structure being left behind, an accident which hastens the progress of the malady;—in as much as absolute certainty of the freedom of internal organs from the disease is unattainable;—in as much as the dormant cancerous diathesis is sometimes roused into activity by removal of a tumour;—in as much as cancers in a state of active growth acquire increased energy of vegetation, if reproduced after extirpation;—and, lastly, in as much as the operation itself has not very unfrequently proved both the occasion and the cause of death; *excision cannot be undertaken without imminent risk of placing the patient in a worse condition than he or she was previously to the use of the knife.* Thirdly: as a corollary from the first and second inferences; and in as much as the disease has unquestionably been cured, or arrested in its progress, by milder measures;—and in as much as the disease does not by any means inevitably and unfailingly run a course destructive of life;—and in as much as quiescent cancers have sometimes been succeeded by most active and virulent growths in consequence of untimely extirpation; and inasmuch as life has sometimes fallen a sacrifice to operations for the removal of putative cancers; *the operation should, as a general rule, be abstained from.*" (pp. 236-7.)

It will, indeed, in our opinion, be a happy day for the honour of surgery and the interests of humanity, when the rash use of the knife is for ever banished to the place of things which are not.

But, setting aside all expectations of cure, may we not operate in the hope of *prolonging the patient's life?* Let the following facts, adduced by Dr. Walshe, answer the question.

In 85 of Benedict's 98 cases of amputation of the breast, death was *hastened* by the operation. From the experience of Dr. Macfarlane, whose operations were executed under the most favorable circumstances, it appears that, of 32 cases, two terminated fatally from the immediate effects of the excision, and reproduction occurred in all the others within the following periods:

| No. of Cases. | Period of Relapse. |
|---|---|
| 9 | Between 6 weeks and 3 months. |
| 13 | Between 3 and 9 months. |
| 4 | Between 9 and 12 months. |
| 3 | Within 24 months. |
| 1 | Nearly 36 months. |

Whence it follows, that the mean period of relapse is something less than eight months and a week; and that the chances are as seven to one that extirpation either proves fatal at once, or is followed by reproduction within twelve months. And that the mean duration of life is not increased by operating is shown by the records collected by M. Leroy d'Etiolles. From the first series of these facts, comparing the duration of life after the sixth year in persons operated on and not operated on, the subjoined table may be formed:

| Total duration of Life from outset of Disease. | Of 1172 Patients not operated on, there reached these periods. | Of 801 Patients operated on, there reached these periods. |
|---|---|---|
| Upwards of 30 years | 18 | 4 |
| From 20 to 30 years | 34 | 14 |
| From 6 to 20 years | 288 | 88 |

Whence it follows, that the commencement of the sixth year of a cancerous disease will be reached by 13·2 per cent. only of those operated on, by 29·7 per cent. of those who have not been submitted to the knife.

It were needless to adduce more proofs of the melancholy fact, that extirpation of cancerous growths with the knife can neither be regarded as a means of cure, nor of prolonging existence; and to our minds the conclusion is irresistible, that, excepting under very peculiar circumstances, such as the threatening of immediate death for incontrollable hemorrhage, or complete occlusion of the rectum in carcinoma of that viscus, the surgeon who cares for his own reputation and the real welfare of his patient should refuse to subject him to the danger and torture of an useless operation.

The other methods of ablation will not detain us. Cures have been effected by the enucleation of tumours by caustics (and for this purpose the chloride of zinc is perhaps the best and safest), and even by the induction of sphacelus by inoculation of the matter of hospital gangrene. But the experiments have not been conducted on a sufficiently large scale to warrant any satisfactory conclusion; and, while better and less hazardous means are in our power, we should hesitate long before advising a course so painful.

Here, for the present, we must pause with the remark, that in all that precedes we have given a mere epitome of the author's views. These seemed to us so just, that we found no ground for cavil or criticism. Our analysis is avowedly meager and dry, but we believe it will be found accurate.

In our next Number we shall hope to accompany our readers through the remainder of this truly valuable work, and help them to form a closer acquaintance with this formidable disease in the various organs and tissues which it infests.

